# CSB affected on the sensitivity of lung cancer cells to platinum-based drugs through the global decrease of let-7 and miR-29

**DOI:** 10.1186/s12885-019-6194-z

**Published:** 2019-10-15

**Authors:** Zhenbang Yang, Chunling Liu, Hongjiao Wu, Yuning Xie, Hui Gao, Xuemei Zhang

**Affiliations:** 1 Institute of Molecular Genetics, College of Life Science, North China University of Science and Technology, Tangshan, China; 2 Hebei Key Laboratory of Basic Medicine for Chronic Disease, School of Basic Medical Sciences, North China University of Science and Technology, Tangshan, China; 3 Department of Pathology, Affiliated Tangshan Renmin Hospital North China University of Science and Technology, Tangshan, China; 4 Institute of Epidemiology, School of Public Health, North China University of Science and Technology, Tangshan, China

**Keywords:** CSB, Let-7, miR-29, Platinum, Lung cancer

## Abstract

**Background:**

Transcription-coupled nucleotide excision repair (TC-NER) plays a prominent role in the removal of DNA adducts induced by platinum-based chemotherapy reagents. Cockayne syndrome protein B (CSB), the master sensor of TCR, is also involved in the platinum resistant. Let-7 and miR-29 binding sites are highly conserved in the proximal 3′UTR of CSB.

**Methods:**

We conducted immunohistochemisty to examine the expression of CSB in NSCLC. To determine whether let-7 family and miR-29 family directly interact with the putative target sites in the 3′UTR of CSB, we used luciferase reporter gene analysis. To detect the sensitivity of non-small cell lung cancer (NSCLC) cells to platinum-based drugs, CCK analysis and apoptosis analysis were performed.

**Results:**

We found that let-7 and miR-29 negatively regulate the expression of CSB by directly targeting to the 3′UTR of CSB. The endogenous CSB expression could be suppressed by let-7 and miR-29 in lung cancer cells. The suppression of CSB activity by endogenous let-7 and miR-29 can be robustly reversed by their sponges. Down-regulation of CSB induced apoptosis and increased the sensitivity of NSCLC cells to cisplatin and carboplatin drugs. Let-7 and miR-29 directly effect on cisplatin and carboplatin sensitivity in NSCLC.

**Conclusions:**

In conclusion, the platinum-based drug resistant of lung cancer cells may involve in the regulation of let-7 and miR-29 to CSB.

## Background

Lung cancer is the leading cause of cancer-related death worldwide. Despite improvements in diagnosis and surgical techniques, platinum-based chemotherapy remains the foundation of treatment for lung cancer, in particular for patients with NSCLC; however, the efficacy is significantly limited. Multiple mechanisms have been causally linked to the platinum drug resistance, such as drug transport, drug detoxification, DNA repair and cell apoptotic [1–3]. To date, the organizing principles of platinum drug resistance are still not fully understood.

Platinum drugs (mainly cisplatin and carboplatin) form several types of DNA adduct lesions including the predominating 1,2-d(GpG) and 1,2-d(ApG) intrastrand crosslinks (90%), followed by 1,3-d(GpNpG) intrastrand crosslinks (5-10%), with minor amounts of 1,2-d(GpC) interstrand and DNA-protein crosslinks (2-5%) [4]. The platinum-DNA intrastrand crosslinks are mainly repaired by NER [5], which also contributes to the removal of platinum-DNA interstrand adducts [6]. NER involves recognition and dual incision of the damage, followed by gap filling [7]. Various reports have convincingly shown that abnormal expression of key genes in the process of NER are highly correlated with platinum drug resistance in a variety of tumor types, particularly testicular, ovarian and NSCLC [8]. For example, ERCC1 is the most promising marker of resistance to cisplatin-based adjuvant therapy and the down-regulation of ERCC1-XPF by siRNA sensitizes lung cancer cells to cisplatin [9].

CSB, the master sensor of TC-NER, is overexpressed in a variety of cancers including lung cancer [10]. CSB plays a prominent role in the removal of both cisplatin-DNA intrastrand and interstrand adducts [6, 11]. Intriguingly, CSB has a large 4337-nucleotide-long 3′untranslated region (UTR), nearly half the length of the messenger RNA, which contains two perfectly conserved miRNA binding sites (let-7 and miR-29) among land vertebrates. Let-7, the well-known tumour suppressor family, is among the most abundantly expressed miRNAs in lung tissue. Global down-regulation of let-7 members is common in lung cancer tissue [12]. The miR-29 family is also down-regulated in lung cancer tissue and the re-expression of miR-29 in lung cancer cells can inhibit tumorigenesis [13]. Due to the evidences that these miRNAs are involved in the lung cancer, it is important to reveal the role of these miRNAs-driven pathway in the process of lung cancer.

In this study, we demonstrated that CSB is overexpressed in NSCLC tissue. We also found that let-7 and miR-29 directly target CSB and regulate the expression of CSB. Furthermore, our data showed that inactivation of CSB could induce apoptosis and increase the sensitivity of lung cancer cells to cisplatin and carboplatin. Our findings support a role for CSB adjuvant therapy as a viable strategy for cisplatin-based chemotherapeutic sensitivity in NSCLC.

## Methods

### Immunohistochemistry

Histopathological evaluation of human lung cancer was performed by experienced board-certified pathologists with HE-stained lung tumor sections. Patient samples were collected at Affiliated Tangshan Renmin Hospital (Tangshan, China). Their general characteristics were collected at the time of tumor sample collection, including gender, age and AJCC (TNM) tumor stage. This study was approved by the ethics committee of North China University of Science and Technology (No. 12-002). For CSB staining, 4-μm thickness sections cut on paraffin-embedded lung tumor samples were deparaffinized, rehydrated and immersed in 3% hydrogen peroxide solution for 10 min to quench endogenous peroxidase activity. After heat-induced antigen retrieval, tissues were blocked with 5% BSA and incubated with CSB primary antibody (Abcam, ab96089) at 1:250. After that, sections were incubated with biotinylated anti-rabbit secondary antibody, avidin-biotin complex and then developed in DAB using a commercial detection kit (ZSGB-BIO, China, PV-8000) according to the manufacturer’s instructions. Image acquisition was performed with Olympus BX63 microscope and a DP80 camera (Olympus). Quantification CSB-positive cells was performed by calculating DAB positive pixels per area and counted by an ImageJ script.

### Cell culture

Human NSCLC cell lines A549, NCI-2030 and NCI-H1975 were purchased from American Type Culture Collection (ATCC). A549 and NCI-H2030 cells were cultured in GibcoTM Roswell Park Memorial Institute 1640 (RPMI 1640) (Life Technologies, Grand Island, NY, USA) and NCI-H1975 cells were cultured in Dulbecco’s Modified Eagle Medium (DMEM). All mediums were supplemented with 10% fetal bovine serum (FBS; Life Technologies, Grand Island, NY, USA) and antibiotics (100 U/ml penicillin and 100 μg/ml streptomycin) in a humidified incubator with 5% CO_2_ at 37 °C.

### Vector cloning

The 3′UTR of CSB was amplified using primers 5′-CAC CTC GAG ACA ACA TTG CTT CCT AAA CTT TCA AG-3′ (XhoI) and 5′-GTA AGC GGC CGC ACT AAG ACA GCT AAG AAG AAA T-3′ (NotI). PCR product was subcloned into the psiCHECK2 reporter vector (Promega, Madison, MI, USA) to generate psiCHECK2-CSB-3′UTR (WT construct). The let-7 or miR-29 binding site in the 3′UTR of CSB on WT construct were mutated by PCR based enzyme synthesis commercially (Synbio Technologies, China) to create MT-let-7 and MT-miR-29 constructs, respectively. The CMV-d2eGFP-cxcr4 vector (Addgene plasmid 21,967) was digested with XhoI and PmeI and ligated a sponge insert (synthesized commercially, Synbio Technologies) containing 10× let-7, miR-29 and CXCR4 bulged binding sites (let-7: AAC TAT ACA AAA CCT ACC TCA, miR-29: TAA CCG ATT TTC TTG GTG CTA, CXCR4: AAG TTT TCA GAA AGC TAA CA, 4 nt-spacer: CCGG) together to generate let-7, miR-29 and CXCR4 sponge.

### siRNA and miRNA mimics transfection

Lung cancer cells were seeded into 6-well or 96- well plates and transfected with 20 nM siRNA or miRNA mimics (GenePharma, China) using Lipofectamine RNAiMAX Transfection Reagent (ThermoFisher Scientific, Grand Island, NY, USA) according to the manufacturer’s instructions. Cells were harvested after 48 h for further analysis. The sequence of siRNA and miRNA are listed below. CSB siRNA (siCSB), 5′-GUG UGC AUG UGU CUU ACG A-3′ (sense); Let-7a mimic, 5′-UGA GGU AGU AGG UUG UAU AGU U-3′ (sense); let-7f mimic, 5′-UGA GGU AGU AGA UUG UAU AGU U-3′ (sense); miR-29a mimic, 5′-UAG CAC CAU CUG AAA UCG GUU A-3′ (sense); miR-29b mimic, 5′-UAG CAC CAU UUG AAA UCA GUG UU-3′ (sense); miR-29c mimic, 5′-UAG CAC CAU UUG AAA UCG GUU A-3′ (sense); control mimic, 5′-UUC UCC GAA CGU GUC ACG UTT-3′ (sense).

### Luciferase assays

To detect the binding of let-7/miR-29 with CSB 3′UTR, psiCHECK2 vector (WT construct, MT-let-7 or MT-miR-29 construct) was transfected into lung cancer cells using Lipofectamine 2000. We also co-transfected WT construct with either let-7 sponge, miR-29 sponge or CXCR4 sponge to A549 cells. Luciferase activities were determined using the Dual-Luciferase Assay System (Promega, Madison, MI, USA) according to the manufacturer’s instructions. The GloMax20/20 Luminometer (Promega, USA) was used to measure fluorescence intensity, followed by a 2-s pre-read delay and a 10-s measurement period. Renilla luciferase activity was used to normalize firefly luciferase activity.

### Western blotting

Western blot analysis was performed using standard methods. CSB protein levels were quantified with whole cell extracts using antibodies against CSB (Abcam, ab96089) and β-actin (Santa Cruz Biotechnology, sc-8342).

### qPCR

Total RNA was isolated from lung cancer cells using Trizol reagent (ThermoFisher Scientific, NY, USA). For both mRNA and miRNA expression analysis, cDNA was prepared from 1 μg RNA using Maxima H Minus First Strand cDNA Synthesis Kit with dsDNase (ThermoFisher Scientific, NY, USA) and oligo (dT)_18_ primer. 20 ng of cDNA was then used for qPCR with the Power SYBR Green PCR Master Mix (ThermoFisher Scientific, NY, USA). The qPCR primers for targeting distinct polyadenylation sites on CSB 3′UTR and apoptosis analysis were list in Additional file 1. Relative expression was determined using the 2^-ΔΔCT^ method.

### Generation of stable cell lines

GV248 short hairpin RNA (shRNA) constructs were synthesized by Genechem (Shanghai, China). The sequence of CSB shRNA and control shRNA are 5′-GTG TGC ATG TGT CTT ACG A-3′ and 5′-TTC TCC GAA CGT GTC ACG T-3′, respectively. GV369 pri-miRNA expression constructs (Genechem, Shanghai, China): let-7f-1 pri-miRNA (forward: 5′-GAG GAT CCC CGG GTA CCG GTT TCT TTC GAA AGA GAT TGT ACT TTC CAT TC-3′; reverse: 5′-CAC ACA TTC CAC AGG CTA GTA CTT GAA CAG AGA AAA TTA AC-3′); miR-29a pri-miRNA (forward: 5′-GAG GAT CCC CGG GTA CCG GTC ATT CCA TTG TGC CTG G-3′; reverse: 5′-CAC ACA TTC CAC AGG CTA GTT GCT TTG CAT TTG TTT TC-3′); control vector (no insert). Lentiviral particles were produced by co-transfection of HEK293 cells with GV248/GV369 vectors and packaging vectors pHelper 1.0 and pHelper 2.0 from Shanghai Genechem. H2030 cells were infected with either shRNA or pri-miRNA lentivirus in 6-well plates and subsequently split into 10-cm dishes 48 h after infection in the presence of 2 μg/ml puromycin for selection over 1 week.

### Drug treatment and CCK analysis

For CCK analysis, lentiviral H2030 cells were treated with either 10-70 μM cisplatin (Sigma-Aldrich, USA) or 80-640 μg/ml carboplatin (Sigma-Aldrich, USA) for 24 h. siRNA and miRNA mimics transfected H2030 cells were treated with either 4-32 μM cisplatin or 30-240 μg/ml carboplatin for 48 h. Four thousand cells per well were plated on a 96-well plate in sextuplicate overnight. After drug treatment, cells were then treated with WST-8 Cell Counting reagent (Dojindo, Japan) for 1 h at 37 °C according to the manufacturer’s protocol. Analysis was performed using the Infinite M200 PRO Microplate Reader (Tecan) with 450 nm absorbance and the survival rate was calculated by normalizing untreated cells to 100%.

### Apoptosis analysis

PE Annexin V Apoptosis detection kit (BD Biosciences, CA) was used to detect apoptosis. Cells were treated with cisplatin (12 μM) or carboplatin (80 μg/ml) for 48 h and were collected by centrifugation, resuspended in 400 μl binding buffer, followed by staining with 5 μl PE Annexin V and 5 μl 7-ADD for 15 min in dark at room temperature. Apoptotic cells were then evaluated by 7-ADD and Annexin V-positive cells on a fluorescence-activated cell-sorting (FACS) flow cytometer (Beckman Coulter, CA).

### Bioinformatics and statistical analysis

To determine the potential miRNAs binding to CSB 3′UTR sequence, we used two commonly online miRNA prediction programs: TargetScan (http://www.targetscan.org/) and miRanda (http://www.microrna.org). The expression of CSB was analyzed using The Cancer Genome Atlas (TCGA) data sets (https://portal.gdc.cancer.gov) and Gene Expression Profiling Interactive Analysis (GEPIA) (http://gepia.cancer-pku.cn/).

### Statistical testing

Data are expressed as means ± SD. Statistical significance was assessed by the Student’s t-test. *P* values less than 0.05 were considered significant.

## Results

### CSB expression is up-regulated in NSCLC

**Fig. 1:**
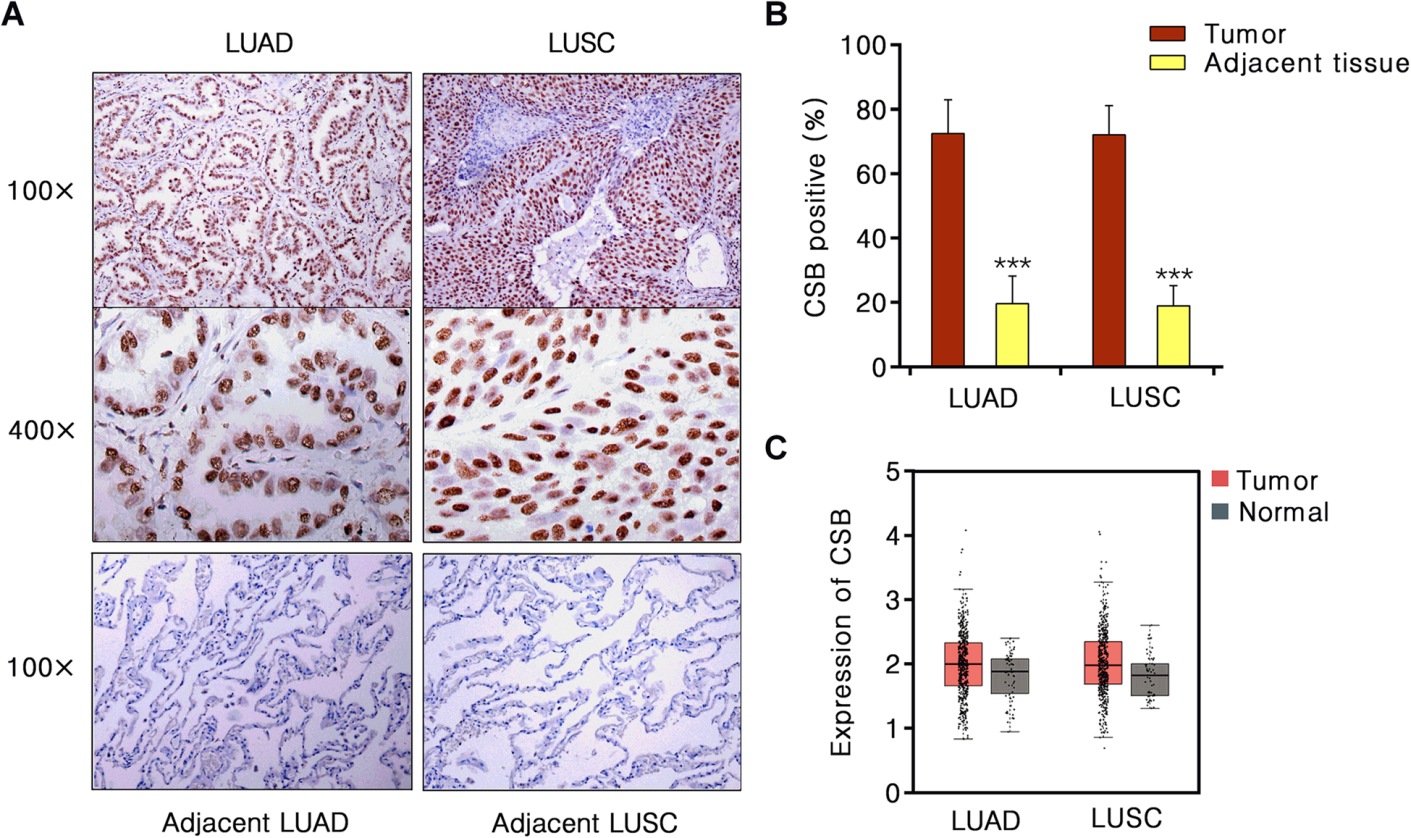
Immunohistochemical visualization of CSB protein in NSCLC and adjacent lung tissues. **a** Representative CSB immunohistochemical staining of human NSCLC and normal lung tissues. **b** Percentage CSB positive LUAD and adjacent normal tissues (*n* = 43); LUSC and adjacent normal tissues (*n* = 43). LUAD, lung adenocarcinoma; LUSC, lung squamous carcinoma. **c** The CSB mRNA level in 483 LUAD tissues and 59 normal tissues, 486 LUSC tissues and 50 normal tissues from GEPIA

To examine the expression of CSB in NSCLC, we performed immunohistochemistry in 43 lung adenocarcinoma (LUAD) samples and 43 squamous carcinoma (LUSC) samples, and their paired adjacent normal tissues. As shown in Fig. 1 a and b, CSB was mainly localized in the nucleus of lung cancer cells. In most lung cancer cases, we observed stronger staining of CSB than in normal tissues. The percentage of positive CSB in LUAD (72.56%) and LUSC tissues (72.09%) were significantly higher than that in paired adjacent normal tissues (19.61 and 18.96%, respectively) (*P* < 0.001). We then analyzed the RNA expression of CSB in lung cancer and normal tissues using the GEPIA (483 LUAD vs 59 normal tissues; 486 LUSC vs 50 normal tissues). The RNA level of CSB in lung cancer tumor tissue was significantly higher than that in normal tissue (Fig. 1c).

### Let-7 and miR-29 8mer binging sites are highly conserved in the proximal 3′UTR of CSB across species

**Fig. 2:**
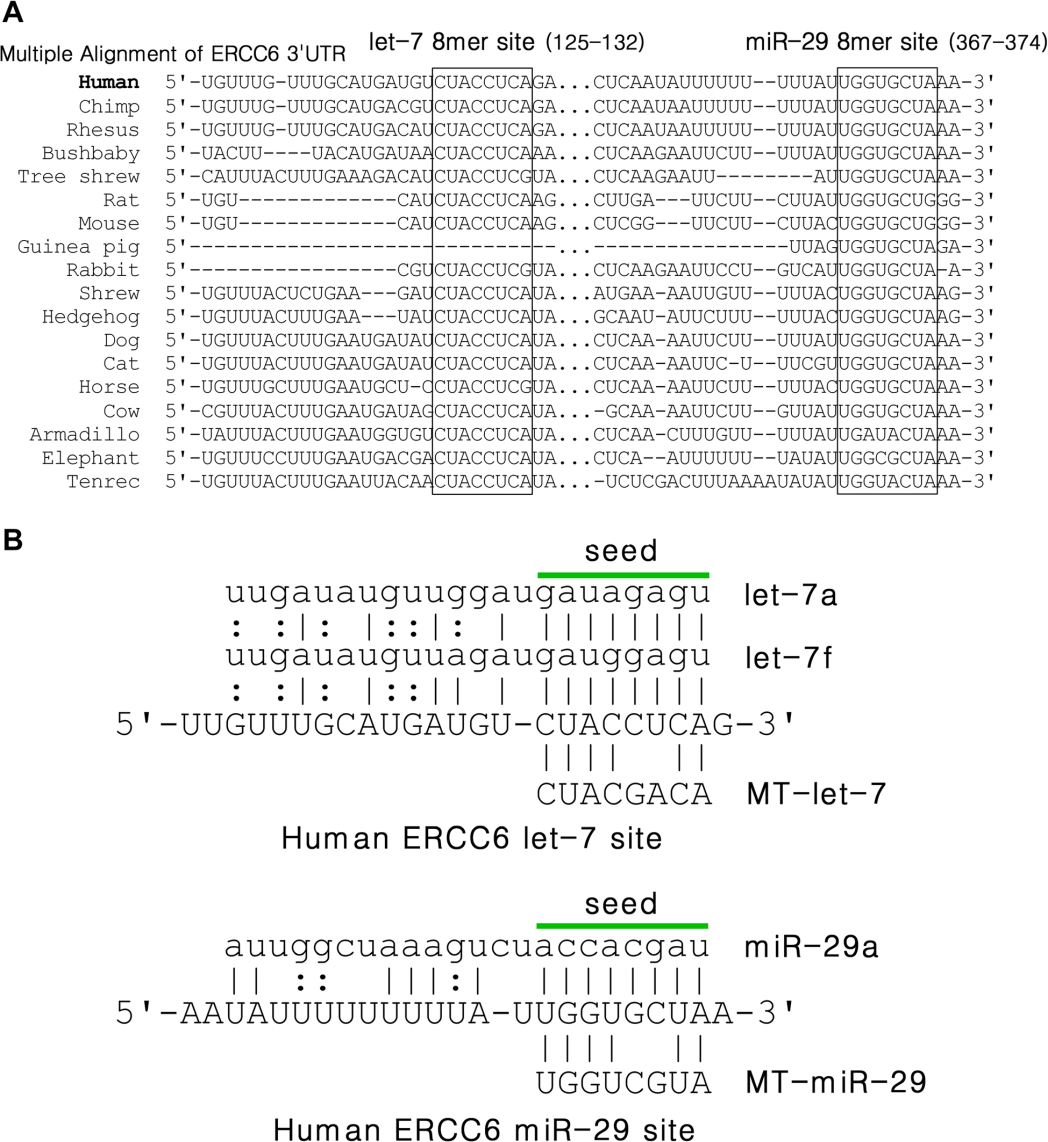
CSB is a highly conserved let-7 and miR-29 target. **a** Alignments of let-7 and miRNA-29 sites in 18 placental mammals CSB 3′UTRs. **b** Predicted binding patterns of let-7and miR-29 with CSB

Based upon the online miRNA target prediction tools, TargetScan and miRnada, two 8mer sites of let-7 (position 125-132) and miR-29 (position 367-374) are highlighted in the 3′UTR transcript of CSB (4337 nt, NM_000124), both residing in more proximal 3′UTR contexts (Fig. 2). Importantly, most of the 3′UTR of CSB is divergent in evolution among land vertebrates; however, the single 8mer sites of let-7 and miR-29 are broadly evolutionarily conserved across nearly all mammalian species.

**Fig. 3:**
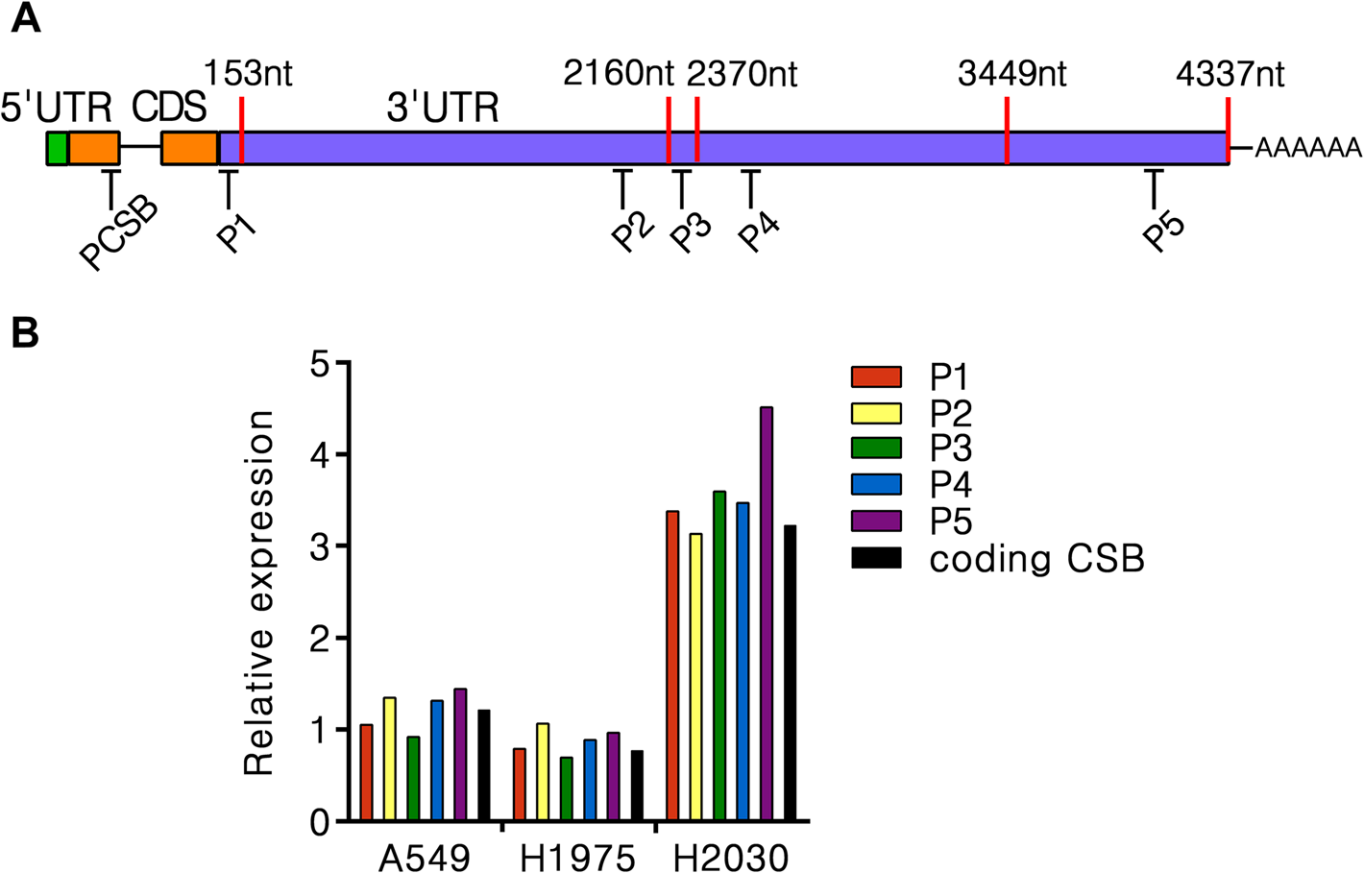
ERCC6 gene expresses the longest known CSB-3′UTR isoform in NSCLC. **a** Schematic of human CSB 3′UTR-APA (alternative polyadenylation), indicating poly(A) sites (red strip) and their approximate location. Specific qPCR strategy amplifying five distinct CSB 3′UTR isoforms used in P1-P5 primer pairs. **b** qPCR analysis of distinct CSB 3′UTR isoforms levels in A549, H1975 and H2030 cells. Values are normalized to GAPDH. Mean of two independent experiments shown

An important consideration in the accurate prediction of miRNA-target interactions is the usage of alternative 3′UTR isoforms by influencing both the presence and scoring of target sites [14]. Some researchers have identified two alternative tandem 3′UTR isoforms of CSB (153 nt and 2160 nt) using Northern blot analysis [15]. Recently, longer tandem 3′UTR isoforms were found by sequencing (2370 nt, CR749388; 3449 nt, ENST00000355832; 4337 nt, NM_000124) (Fig. 3a, Additional file 1). Notably, each of these isoforms contains a canonical poly (A) signal (PAS) located in 35 nt upstream of their corresponding poly (A) sites. Since the putative 8mer site of let-7 is located in the shortest 3′UTR isoform of CSB, all five identified 3′UTR transcripts are potentially subject to the let-7-mediated regulation. In contrast, the putative 8mer site of miR-29 is located beyond the shortest one but in the second one, so the longer isoforms of CSB may affect the potential regulation by miR-29. In present study, we examined the relative levels of these 3′UTR isoforms in lung cancer cells by specific qPCR analysis and observed a similar CSB expression in all transcript isoforms in A549, H1975, and H2030 cells (Fig. 3b). This result indicates that the longest known 4337 nt 3′UTR isoform might be prevalent in lung cancer cells and is also affected by the potential let-7 or miR-29 mediated regulation.

### Let-7 and miR-29 directly target to the 3′UTR of CSB

**Fig. 4:**
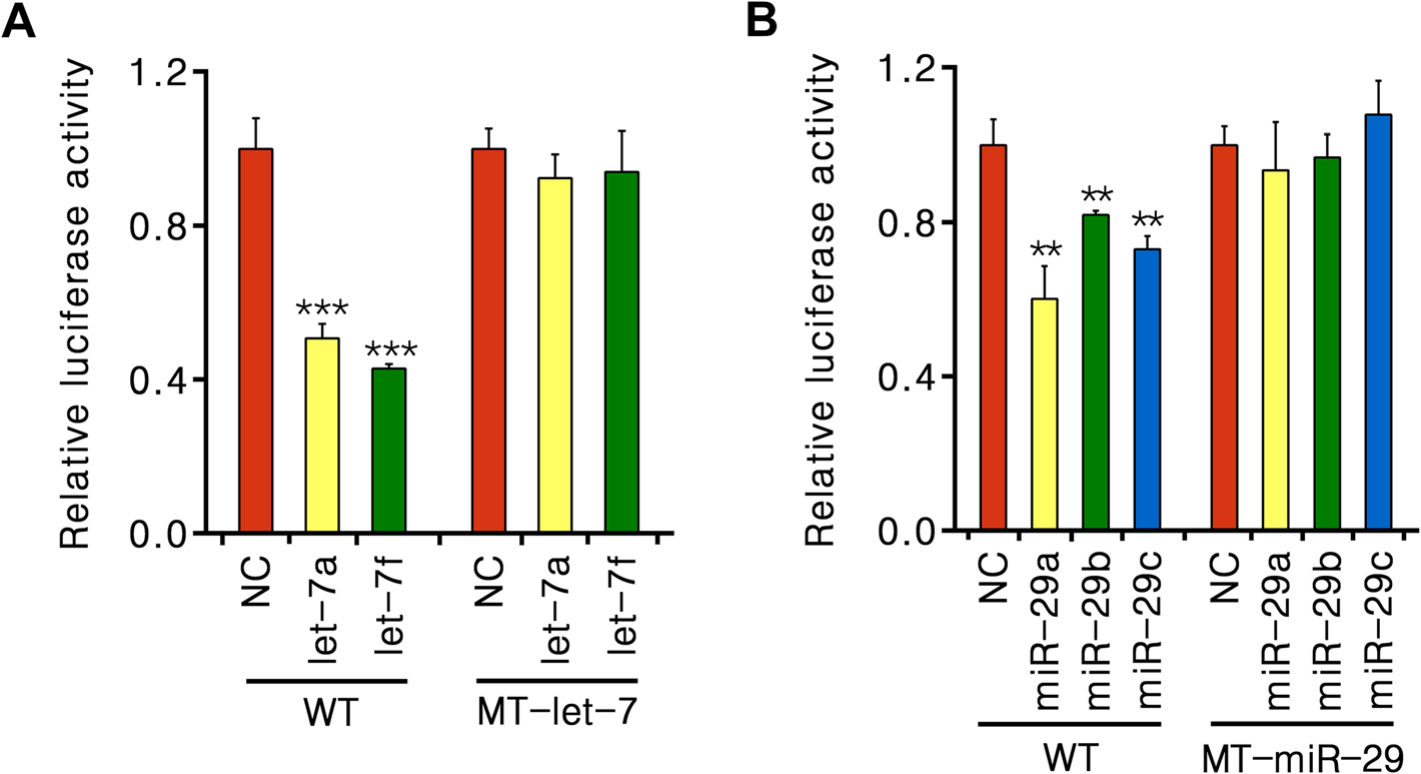
Let-7 and miR-29 directly target to CSB 3′UTR. **a** and **b** Relative fluorescence ratio in A549 cells co-transfected with miRNA mimics and either the wild-type (WT) or let-7/miR-29-mutant CSB 3′UTR (MT-let-7/MT-miR-29) constructs. Values are normalized to NC miRNA mimic of either WT or MT-let-7/MT-miR-29 construct. **P < 0.01, ***P < 0.001

To determine whether let-7 family and miR-29 family directly interact with the putative target sites in the 3′UTR of CSB, we generated three psiCHECK2-CSB-3′UTR luciferase reporter constructs with WT, MT-let-7 or MT-miR-29. We then co-transfected each construct with either let-7/miR-29 mimics or scrambled mimics into A549 cells. Representative let-7a and let-7f miRNAs highly expressed in A549 cells were used for further analysis [16]. Luciferase reporter gene analysis revealed that the relative Renilla luciferase activity of WT was reduced about 55% in response to additional let-7a or let-7f (*P* < 0.001) and 40% in response to additional miR-29a, 19% to miR-29b, 28% to miR-29c (*P* < 0.01), whereas both MT-let-7 and MT-miR-29 showed no change of luciferase activity (Fig. 4).

### Endogenous let-7 and miR-29 in lung cancer cells confirm the miRNA-mRNA interaction on CSB targets

**Fig. 5:**
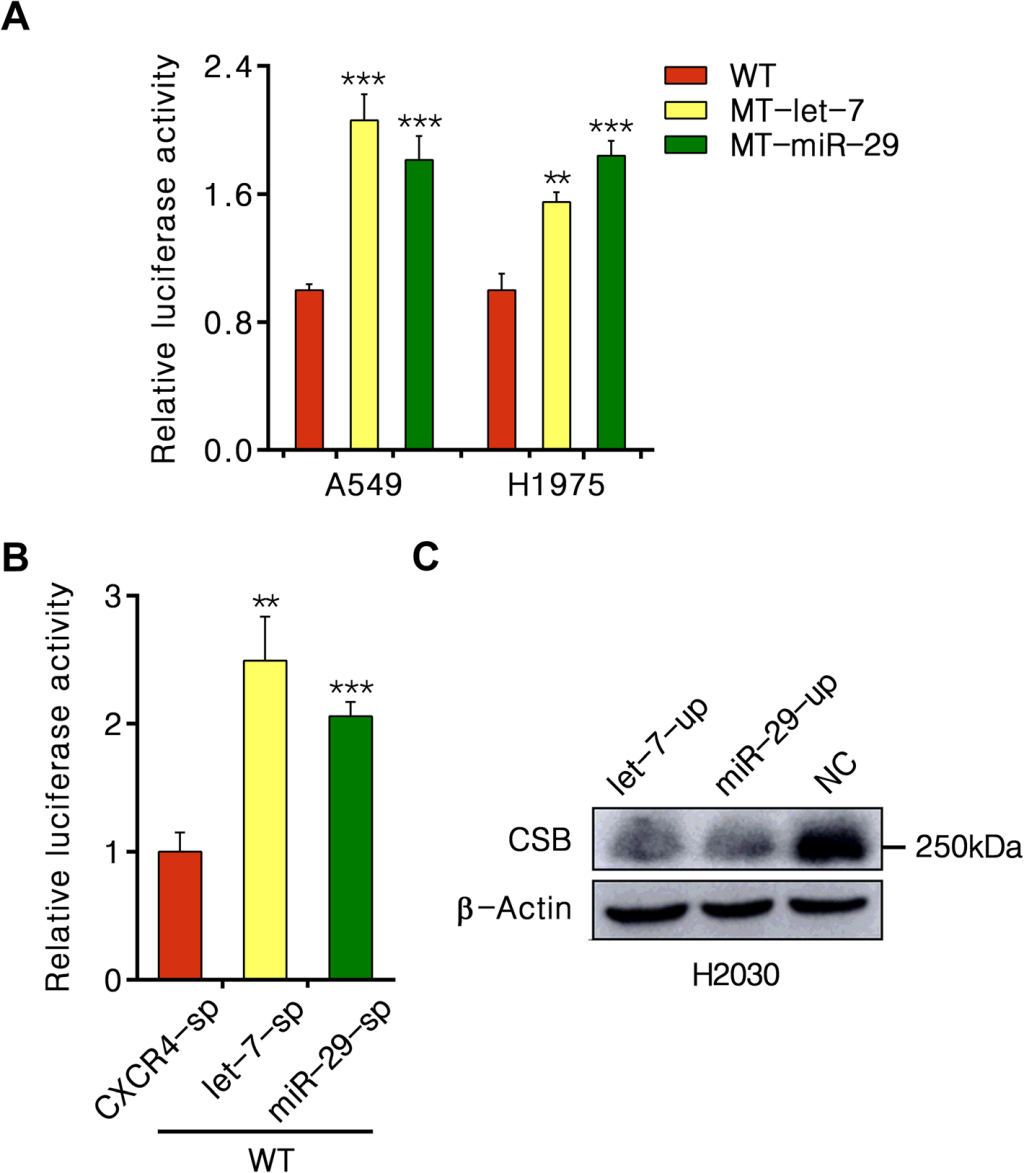
CSB activity is regulated by endogenous let-7 and miR-29. **a** Relative fluorescence ratio in cells transfected with WT, MT-let-7 and MT-miR-29 constructs respectively. **b** Relative fluorescence ratio in A549 cells co-transfected with WT and let-7 sponge (let-7-sp) miR-29 sponge (miR-29-sp) or CXCR4 sponge (CXCR4-sp) constructs. Values are normalized to CXCR4-sp construct. **P < 0.01, ***P < 0.001. **c** Immunoblot for CSB in H2030 cells infected with lentiviral let-7f-1 and miR-29a pri-miRNAs

As with the lung cancer cells highly expressing native let-7 and miR-29, we examined the interaction of endogenous let-7 and miR-29 with CSB targets by directly transfecting each psiCHECK2 construct into lung cancer cells (A549 and H1975). We observed a striking suppression of WT construct compared to MT-let-7/MT-miR-29 in both cell lines (*P* < 0.01) (Fig. 5a).

To elucidate the ability of endogenous let-7 and miR-29 on CSB targets more broadly, we performed miRNA knockdown in A549 cell. Considering the presence of various let-7 and miR-29 isoforms in human lung cancer cells, we used miRNA sponges, which can function as sinks for pools of active miRNAs, liberating transcripts targeted by that set of miRNAs, to dilute all endogenous let-7 or miR-29 members in A549 cell specifically. As shown in Fig. 5b, the designed let-7 and miR-29 sponges efficiently blocked their function. The suppression of WT activity by endogenous let-7/miR-29 can be robustly reversed by let-7/miR-29 sponge (*P* < 0.01), but not by CXCR4 sponge.

### Increase in let-7 and miR-29 abundance in NSCLC cells suppress endogenous CSB expression

To test the effect of let-7 and miR-29 on the expression of CSB, we generated lentiviral let-7f-1 and miR-29a pri-miRNA expression constructs and a control construct in which the pre-miRNA stem loop was deleted. After transfecting each construct into H2030 cells, we found that the corresponding expression of let-7f-1 and miR-29a pri-miRNAs were increased over 100-fold compared to the control. As shown in Fig. 5c, CSB protein level was dramatically decreased with expression of either let-7f or miR-29a construct, suggesting the endogenous CSB expression can be suppressed by let-7 and miR-29.

### Down-regulation of CSB increases the sensitivity of NSCLC cells to cisplatin and carboplatin drugs

**Fig. 6:**
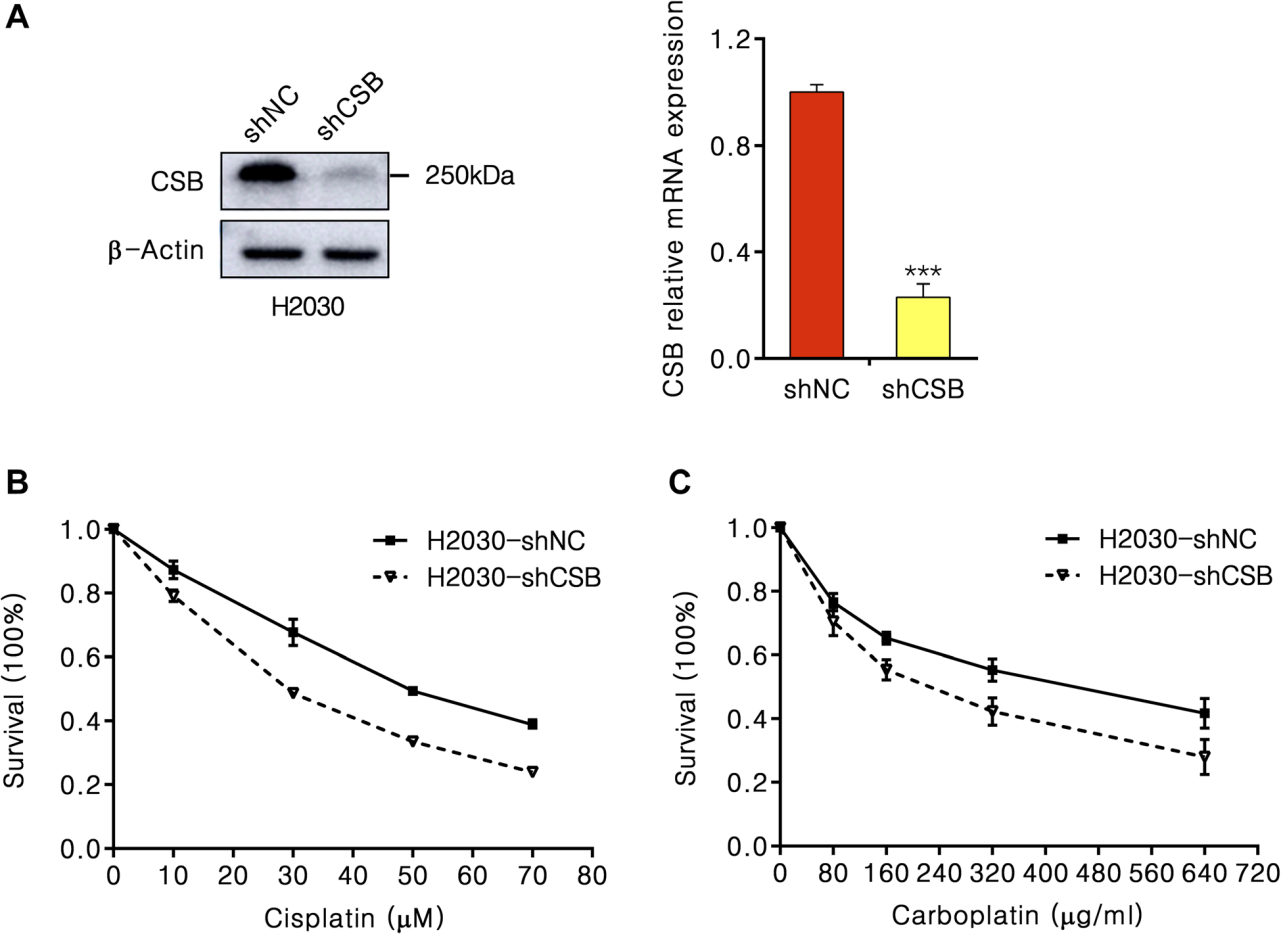
Short hairpin knockdown of CSB sensitizes H2030 cells to cisplatin and carboplatin. **a** Immunoblot for CSB in H2030 cells infected with CSB targeting lentiviral shRNAs (**a**) and corresponding qPCR analysis of relative CSB levels (**b**). **b** and **c** Cell growth analysis of H2030-shCSB and H2030-shNC cells with 10-70 μM cisplatin or 80-640 μg/ml carboplatin for 24 h

To assess the effect of CSB on the development of NSCLC treated by cisplatin or carboplatin, we transfected lentiviral short hairpin RNA (shRNA) construct to H2030 cells to knockdown CSB and observed a striking reduced CSB protein levels (*P* < 0.001) (Fig. 6a). We then tested the cell viability after CSB knockdown in response to cisplatin and carboplatin. Significant growth suppression was observed in CSB-knockdown H2030 cells treated by cisplatin or carboplatin (Fig. 6b, c). In response to cisplatin and carboplatin, the 50% inhibitory concentrations (IC_50_) value was decreased 6-fold and 4-fold after inhibiting CSB. These results indicate that CSB promotes the formation of cisplatin and carboplatin resistance in NSCLC, with potential implications for lung cancer chemotherapy.

### CSB inhibition induces apoptosis to sensitize platinum resistant NSCLC cells

**Fig. 7:**
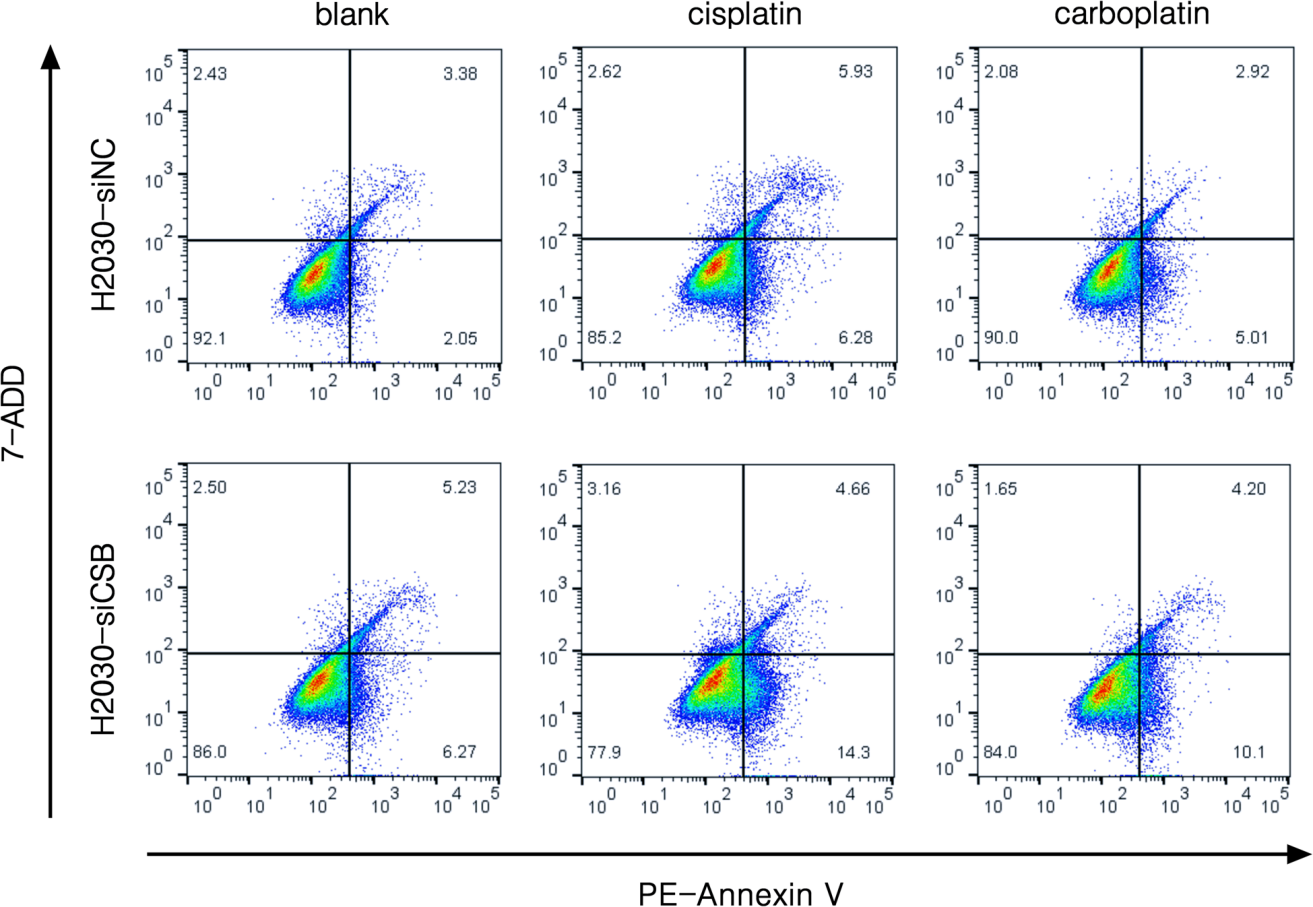
Inhibition of CSB induces NSCLC cell apoptosis. The effect of CSB siRNA knockdown on apoptosis of H2030 cells treated with 12 μM cisplatin or 80 μg/ml carboplatin for 48 h

To better understand the physiological role of CSB expression in NSCLC cells, we examined apoptosis activity in H2030 cells treated with CSB siRNA and control siRNA by Flow Cytometer. As shown in Fig. 7, the overexpression of CSB in lung cancer cells treated with cisplatin and carboplatin could induce apoptosis and the knockdown of CSB by siRNA significantly increased apoptosis.

### Let-7 and miR-29 sensitize NSCLC cells to cisplatin treatment

**Fig. 8:**
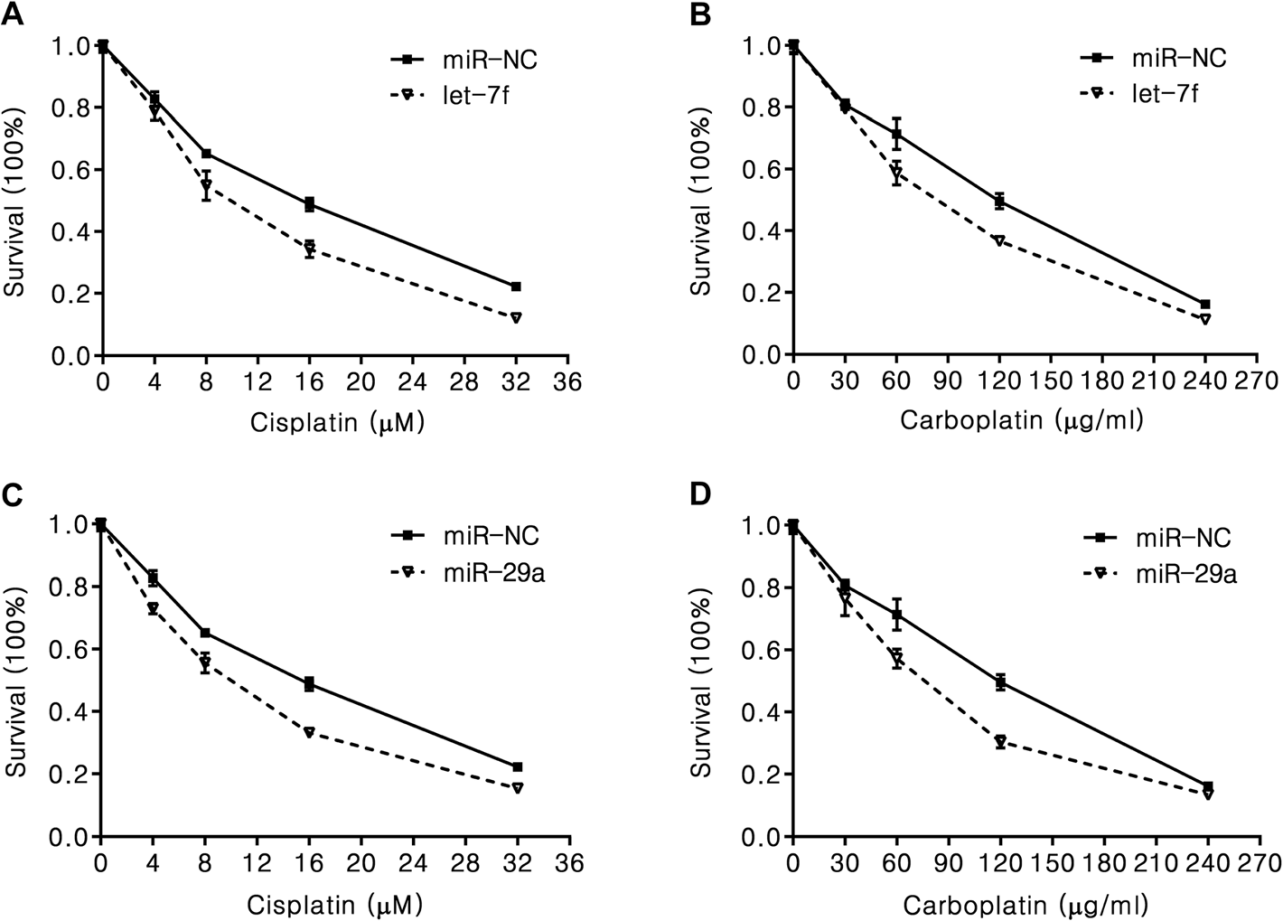
Let-7 and miR-29 sensitize H2030 cells to cisplatin and carboplatin. **a–d** Cell proliferation analysis of let-7f and miR-29a or miR-NC transfected H2030 cells with 4-32 μM cisplatin or 30-240 μg/ml carboplatin for 48 h

**Fig. 9:**
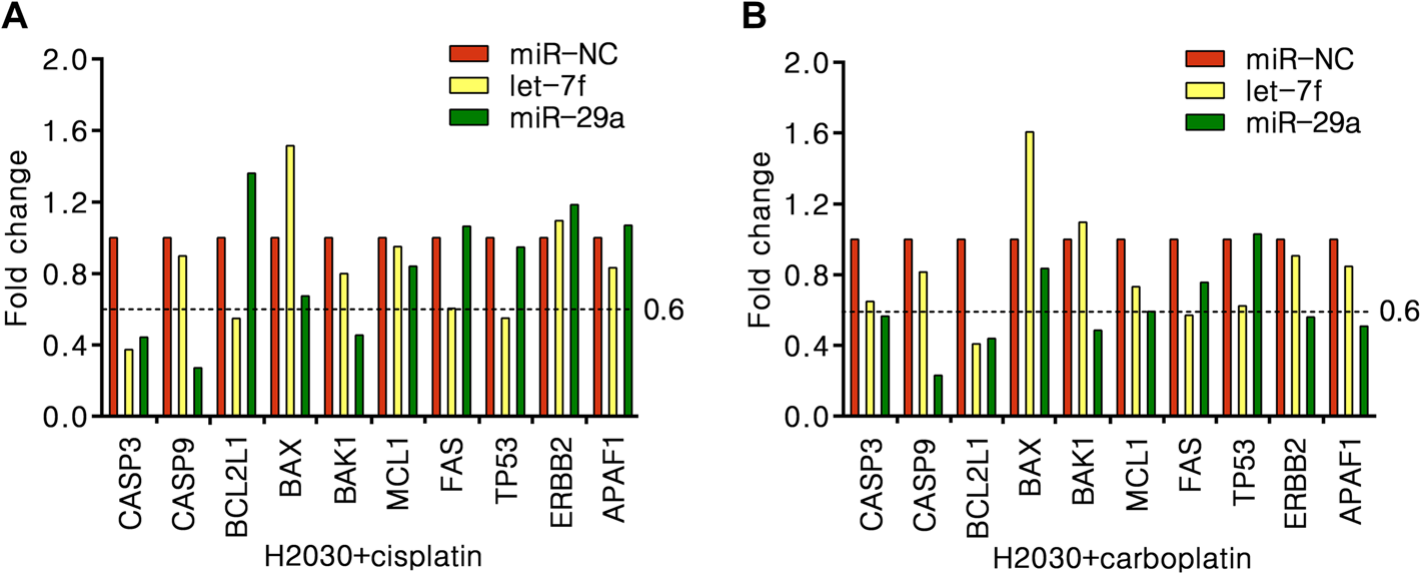
Let-7 and miR-29 effect on the expression of apoptosis genes in platinum-drug treated NSCLC cells. **a** H2030 cells treated with 12 μM cisplatin for 48 h. **b** H2030 cells treated with 80 μg/ml carboplatin for 48 h

Based on the above findings and due to the ability of let-7 and miR-29 to act as tumor suppressors, we then examined whether let-7 and miR-29 can directly operate as contributors for cisplatin sensitivity in NSCLC cells. After transfected let-7f or miR-29a mimics into H2030 cells, we detected the cell proliferation by CCK analysis. We observed that both miRNAs effect on the cell proliferation in either cisplatin or carboplatin treated group (Fig. 8). To examine the transcriptional activity of apoptosis target genes, we performed qPCR in let-7f, miR-29a or miR-NC mimics transfected H2030 cells with cisplatin and carboplatin treatment. The results showed that the RNA levels of a panel of apoptosis target genes have robust changed in either cisplatin/carboplatin treated both miRNAs groups compared to control groups (Fig. 9). The result suggested that both let-7 and miR-29 may directly effect on cisplatin/carboplatin sensitivity in NSCLC.

## Discussion

In this study, we observed CSB is overexpressed in NSCLC regardless of type of histology. We also found that the transcriptional activity of CSB could be suppressed by global decreased let-7 and miR-29 and promoted by additional let-7 or miRNA-29 in lung cancer cells. After sequestering endogenous let-7 and miR-29 miRNAs, we observed the up-regulation of CSB luciferase activity. The apparent CSB protein occurs in a size of approximately of 250 kDa (CSB monomer of 168 kDa) in H2030 cells, consistent with the reported model of CSB wrapping on average 125 bp of DNA around its surface, suggesting strong functional chromatin-remodeling activity of CSB [17].

CSB is a SWI2/SNF2-like DNA-dependent ATPase that can wind DNA [17] and remodel chromatin [18, 19]. CSB also play important roles in the process of homologous recombination repair (HR) [20, 21], base excision repair (BER) [22, 23], transcription [24] and mitochondrial function [25–27]. Importantly, CSB is overexpressed in a variety of cancer cells including lung cancer and promotes tumor growth, predicting its enhanced repair capacity to cisplatin. Let-7, the well-known tumor suppressor family, is among the most abundantly expressed miRNAs in lung tissue. Global down-regulation of let-7 members is common in lung cancer and has a causative role in the pathogenesis and progression of lung cancer [12].

CSB has been shown to establish a critical negative feedback loop with tumor suppressor p53, which maintains a balance between cellular aging and cancer susceptibility [28]. Interestingly, both let-7 [29–31] and miR-29 [32, 33] seem to form a positive feedback loop with p53 via regulation of upstream regulators of p53 that reinforces its effector functions, such as apoptosis and senescence. Connecting these observations with our results, we presume that let-7 and miR-29-mediated regulation is also a pivotal part of CSB-p53 feedback loop, robustly ensuring the fine CSB-p53 interaction and balance between cellular aging and tumorigenesis.

Our findings also suggested that CSB knockdown could sensitize H2030 cells to platinum-based drugs and induce a potent antiproliferative effect. In consistent with our finding, the suppression of CSB activity has been proved to give rise to cisplatin sensitivity in ovarian, prostate and colon cancer cells [34, 35]. Recently, two reports have shown that genetic polymorphisms of ERCC6 could affect sensitivity of NSCLC patients to platinum-based chemotherapy, which confirming the important role of CSB in predicting chemotherapy sensitivity and toxicity [36, 37]. Although interstrand crosslinks (ICLs) account for the minority of all types of cisplatin-damages, they are considered extremely toxic by blocking fundamental cellular processes such as replication and transcription and further leading to cell death or genome instability. Researchers also provided many compelling evidences that increased repair of ICLs were highly correlated to the cisplatin resistance [38–40]. CSB plays a critical role in unhooking cisplatin-induced ICLs and restarting transcription in a replication-independent, transcription-associated mechanism, implying its potential as a therapeutic target [6, 11]. Notably, despite of TCR, CSB also functions in HR and BER pathways specifically, both of which are implicated in the removal of cisplatin induced ICLs [41, 42].

The deregulation of non-coding RNAs, especially miRNA, has emerged as an important mechanism of cisplatin resistance implicated in numerous cancers including lung cancer [43–45]. The ability of a single miRNA to affect the expression of multiple proteins has led to increased interest in miRNAs as mediators of the cellular response to DNA-damaging agents. We found that both let-7 and miR-29 could enhance the sensitivity of cisplatin and carboplatin in H2030 cells with suppression of CSB. This finding is consistent with previous year’s reports that let-7 could enhance the sensitivity of glioma, medulloblastoma and esophageal carcinoma to cisplatin treatment and miR-29 modulate the chemosensitivity to cisplatin in nasopharyngeal carcinoma and lung cancer cells [46–50]. Further studies are still required to fully determine whether this effect is common to other NSCLC cells.

## Conclusion

**Fig. 10:**
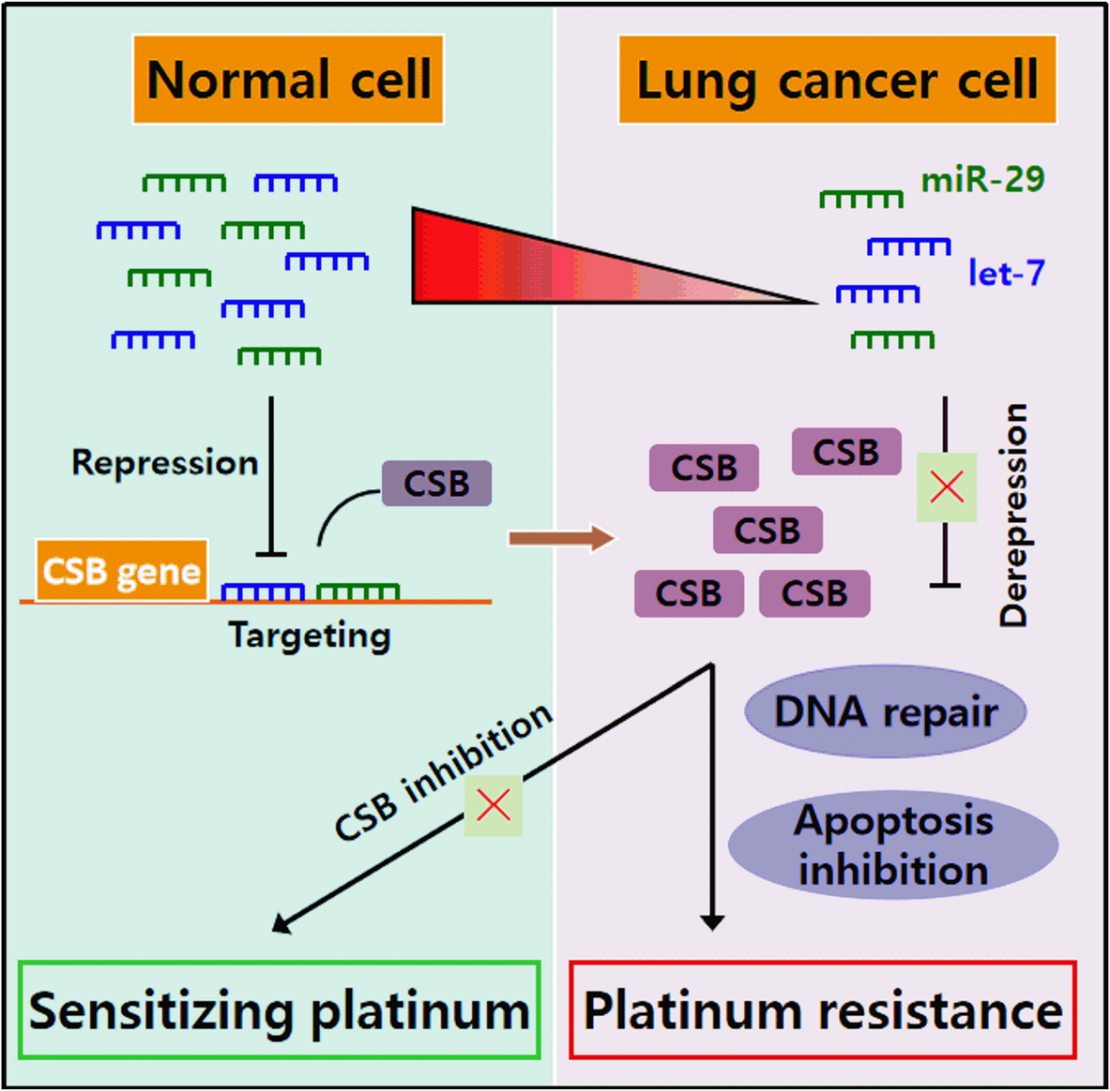
Schematic diagram summarizing the signaling pathway

The platinum-based drug resistant of lung cancer cells may involve in the regulation of let-7 and miR-29 to CSB (Fig. 10).

## Supplementary information

**Additional file 1.** Primers used for the qPCR analysis of mRNAs, related to Figs. 3, 6 and 9.

## Data Availability

The datasets used and/or analyzed during the current study are available from the corresponding author on reasonable request.

## Abbreviations

3′UTR: 3′untranslated region
APA: Alternative polyadenylation
BER: Base excision repair
CSB: Cockayne syndrome protein B
CXCR4: C-X-C motif chemokine receptor 4
DNA: Deoxyribonucleic acid
ERCC1: Excision repair cross-complementation group1
GEPIA: Gene Expression Profiling Interactive Analysis
HR: Homologous recombination repair
ICLs: Interstrand crosslinks
LUAD: Lung adenocarcinoma
LUSC: Lung squamous carcinoma
miRNA: MicroRNA
NC: Negative control
NER: Nucleotide excision repair
NSCLC: Non-small cell lung cancer
PAS: Poly (A) signal
qPCR: Quantitative polymerase chain reaction
TCGA: The Cancer Genome Atlas
TCR: Transcription-coupled nucleotide excision repair
XPF: Xeroderma pigmentosum group F
