# Supplementary material for: CSB affected on the sensitivity of lung cancer cells to platinum-based drugs through the global decrease of let-7 and miR-29
Source: BMC Cancer. 2019 Oct 15;19:948. doi: 10.1186/s12885-019-6194-z (PMC6792260; doi:10.1186/s12885-019-6194-z)
Supplement: Supplementary file 1 — Additional file 1. Primers used for the qPCR analysis of mRNAs, related to Figs. 3, 6 and 9. [file 12885_2019_6194_MOESM1_ESM.doc]

Additional file 1: Primers used for the qPCR analysis, related to Fig. 3, 6, 9.

|  | **Forward** | **Reverse** |
| --- | --- | --- |
| CSB-3'UTR-P1 | GTGTCTTCCGAGAACTATTGAG | GCAGCCAACTTCCATTGAC |
| CSB-3'UTR-P2 | GGTATAGCCTCCTCTTCAAGT | CACGGTAACAATGTGTCAGAT |
| CSB-3'UTR-P3 | CACCATCTTGCACCTGTTC | GCAGTCTGTGTCATGTGATT |
| CSB-3'UTR-P4 | GCACTCTCCTTGACATTCC | CCTTCCTCAACACATCACTT |
| CSB-3'UTR-P5 | CAGAATGGCAATGTGAAGG | TTCCAGTGGCATTAAGTGTT |
| CSB-CDS | GGAACAGAGCAATGACGAT | GCCTCCACCAGTACATAATC |
| GAPDH | CTGGGCTACACTGAGCACC | AAGTGGTCGTTGAGGGCAATG |
| APAF1 | GCCAAGCAGGAGGTCGATAATG | GACCATCCTCAGAAAAGCAGGC |
| BAK1 | TTACCGCCATCAGCAGGAACAG | GGAACTCTGAGTCATAGCGTCG |
| BAX | TCAGGATGCGTCCACCAAGAAG | TGTGTCCACGGCGGCAATCATC |
| BCL2L1 | GCCACTTACCTGAATGACCACC | AACCAGCGGTTGAAGCGTTCCT |
| CASP3 | GGAAGCGAATCAATGGACTCTGG | GCATCGACATCTGTACCAGACC |
| CASP9 | GTTTGAGGACCTTCGACCAGCT | CAACGTACCAGGAGCCACTCTT |
| ERBB2 | GGAAGTACACGATGCGGAGACT | ACCTTCCTCAGCTCCGTCTCT |
| FAS | GGACCCAGAATACCAAGTGCAG | GTTGCTGGTGAGTGTGCATTCC |
| MCL1 | CCAAGAAAGCTGCATCGAACCAT | CAGCACATTCCTGATGCCACCT |
| TP53 | CCTCAGCATCTTATCCGAGTGG | TGGATGGTGGTACAGTCAGAGC |
